# Effect of Ambient PM2.5-Bound BbFA and DahA on Small Airway Dysfunction of Primary Schoolchildren in Northeast China

**DOI:** 10.1155/2019/2457964

**Published:** 2019-09-24

**Authors:** Zhen Kang, XiaoBo Liu, Chao Yang, Cheng Wang, XinXiuNan Miao, XiaoLin Na

**Affiliations:** ^1^Department of Environmental Hygiene, Public Health College, Harbin Medical University, Harbin, China; ^2^Harbin Center for Disease Control and Prevention, Harbin, China

## Abstract

Given the lack of research on the schoolchildren exposure to PM2.5-bound PHAs in northeast China, we investigated the effects of exposure to ambient benzo[b]fluoranthene (BbFA) and dibenz[a,h]anthracene (DahA) bound to PM2.5 on pulmonary ventilation dysfunction (PVD) and small airway dysfunction (SAD). PM2.5 samples at two schools (A and B) were collected, and the concentrations of PM2.5-bound 4–6-ring PAHs were analyzed. PVD and SAD were evaluated by pulmonary function tests in 306 students while urinary MDA and CRP levels were measured. The results confirmed that ambient PM2.5-bound 4–6-ring PHA levels were significantly higher and the PVD and SAD incidence in schools A and B were increased during the heating season. We found that PM2.5-bound BbFA, BkFA, BaP, and DahA levels were only correlated with SAD in schoolchildren; the correlation coefficients of BbFA and DahA were the highest effect estimates, possibly due to altered MDA levels. Therefore, this research enables us to better understand the effects of exposure to ambient PM2.5-bound PHAs on pulmonary function parameters. Our results also showed that identification of hazardous PM2.5-bound BbFA and DahA to health is crucial for preventing the respiratory-related diseases.

## 1. Introduction

The growing rate of industrialization, economy, and population has led to contamination and deterioration of the environment. A wide array of epidemiological studies have linked particular matter (PM) exposure to a variety of adverse health effects, including increased frequency and severity of cardiovascular and respiratory diseases [1–4].

Harbin is a cold region in northern China with four distinct seasons. The winter is cold and long. The concentration of PM in the air during the heating season is far higher than during the nonheating season. Several recent studies have found that airway inflammation might exert a critical role in the development of PM2.5-related respiratory diseases [5, 6]. PM2.5 is a complex mixture of various organic chemical substances and its toxicity changes with its composition [7, 8]. PAHs are important constituents of PM2.5 and PM10, both of which have been linked to respiratory health owing to their potential for causing oxidative stress and cytotoxicity [9], inducing an inflammatory response, and impairing immunological function [10–12]. Accumulating evidence suggests that children are more susceptible to the detrimental effects of environmental PAHs [13], including asthma symptoms [14], respiratory diseases [15, 16], and cognitive development [17]. Therefore, the identification of hazardous PM2.5 components to health is crucial for the implementation of efficient air pollution control strategies.

Currently, the existing evidence on the associations between PM2.5-bound PAHs and respiratory diseases has been limited to the various constituents and PAH metabolites analysis [18, 19]. It remains to be determined which kind of PM2.5-bound PAH constituents has the highest impact on pulmonary function parameters. Our aim was to delineate the role of different PM2.5 constituents, including the main 4–6-ring PAHs, on the incidence of pulmonary ventilation dysfunction (PVD) or small airway dysfunction (SAD) in schoolchildren and analyze the correlations between PM2.5-bound PAH constituents and inflammatory response or oxidative stress in the subjects.

## 2. Methods

### 2.1. Study Population

**Figure 1:**
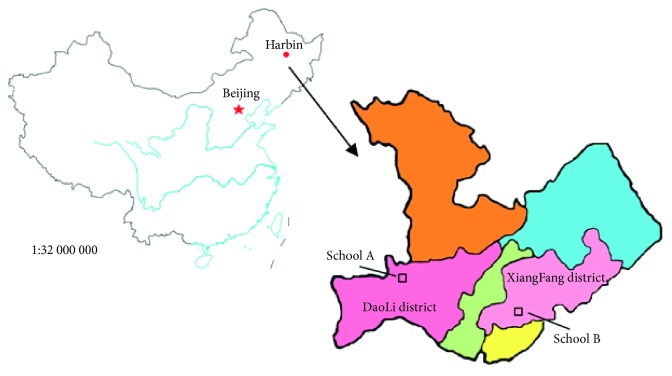
Location of the two primary schools (schools A and B) in Harbin included in this study.

Harbin is in the northeast of China. The winter is long, cold, and dry, and the heating season is from November to March of the following year. The study included students from primary schools located in DaoLi (school A) and XiangFang (school B) districts in Harbin. DaoLi, which is adjacent to the Songhuajiang coast, is the commercial and cultural center of Harbin and has relatively good air quality. In contrast, XiangFang is located in an industrial zone that has highly polluted air according to the Harbin City Environmental Protection Bureau (Figure 1).

We planned to test 320 schoolchildren; however, for various reasons, our dataset ultimately included complete pulmonary function data for only 304 children. Schoolchildren who were in grades 3 to 5 were eligible for inclusion in the study. Schoolchildren with respiratory conditions were excluded. Children's respiratory conditions were determined by the responses to questionnaires, categorized as follows: (a) respiratory diseases including colds and tonsillitis, bronchitis, pneumonia, asthma, rhinitis, or other respiratory diseases; (b) symptoms of respiratory diseases including cough, expectoration, sore throat, and fever within one week before the investigation. The parents of the examined students provided written, informed consent for the participation of their child in the study and filled out questionnaire.

### 2.2. Evaluation of Lung Function

A multifunctional spirometer HI-801 (CHEST M.I., Tokyo, Japan) was used for lung function tests; forced vital capacity (FVC), forced expiratory volume in the first second (FEV1), peak expiratory flow (PEF), and forced expiratory flow at 25% and 75% of vital capacity (FEF25 and FEF75) were measured according to the manufacturer's instructions. All lung function data are expressed as a percentage of the predicted values. Specifically, lung function parameters were measured in a seated position after at least 5 min of rest. The spirometry test was performed by trained professional medical staff. Body weight and height were measured during the medical examination using calibrated measuring instruments. The tests were performed twice on all participating children at the same time of day, between 9:00 and 15:00 in June and December of 2017.

### 2.3. Calculation of Predicted Values

According to the equation for predicting lung function in children and adolescents of Han ethnicity in Heilongjiang Province [20], sex, age, height, and weight were substituted into the regression equation to calculate predicted values. We divided actual value by predicted value to obtain a percentage that was used for statistical analysis.

#### 2.3.1. Assessment of Lung Dysfunction

PVD was measured according to the American Thoracic Society/European Respiratory Society 2005 guidelines on pulmonary function testing as the percentage of predicted FEV1 (FEV1%), with a value <80% determined as PVD. A diagnosis of SAD was made when lung ventilatory function indicators such as FVC, FEV1, and FEV1/FVC were normal (>80% of the predicted value) and FEF75 and FEF25 were <65% of the predicted values [21].

### 2.4. Exposure Assessment

PM2.5 samples were collected from the 10^th^ and 16^th^ of each month in 2017 at the two schools. Samples were collected using a high-volume air sampler (HY-100WS; HengYuan Technology Development Co., Qingdao, China) that was affixed to the school's roof at a height of approximately 10 m, away from heavy traffic and sources of pollution. The pumps were set at an airflow of 100 L/min for PM2.5, with continuous sampling over 24 h. All filters were pre- and postconditioned for 24 h at 25°C ± 1°C and (50 ± 5) % RH, weighed, and stored in a freezer at −20°C until extraction and analysis. Ambient concentrations of PM2.5-bound PAH456 were measured as previously described [22]; these included fluoranthene (FLA), chrysene (CHR), pyrene (PY), benz[a]anthracene (BaA), benzo[a]pyrene (BaP), benzo[b]fluoranthene (BbFA), benzo[k]fluoranthene (BkFA), benzo[ghi]perylene (BghiP), indeno [1,2,3-cd]pyrene (IcdP), and dibenz[a,h]anthracene (DahA). These PAHs represent semivolatile forms of the original 16 priority PAHs; CHR, BaA, BaP, BbFA, BkFA, IcdP, and DahA have been classified as carcinogenic by the U.S. Environmental Protection Agency.

### 2.5. Biochemical Analyses

Urinary C-reactive protein (CRP) concentration was measured by quantitative sandwich enzyme immunoassay assay using a commercial kit (Boster Biological Technology Co., Ltd, Wuhan, China) according to the manufacturer's instructions. Malondialdehyde (MDA) level was measured by an enzymatic method using a kit (Nanjing Jiancheng Bioengineering Institute, Nanjing, China) according to the manufacturer's instructions.

### 2.6. Data Analysis

Descriptive statistics were used to characterize the distributions of ambient PM2.5-bound 4–6-ring PAHs. Prior to analysis, the Kolmogorov–Smirnov test was used to establish the normality of the data. Natural log transformation was applied when the data showed a skewed distribution. Geometric mean (GM) concentrations were used to represent the tendency of ambient PM2.5-bound PAH456 concentrations. Differences in demographic characteristics between the two schools were evaluated with Student's *t*-test in terms of sex, age, height, weight, and urinary CRP and MDA concentrations, and the chi-square test was used for the following parameters: smoking parents, pet in the home, use of an air purifier, distance of outdoor vehicular traffic, and occurrence of PVD or SAD. The differences in % predicted values of lung function parameters between groups were compared by analyses of variance. Binary logistic regression was used to evaluate the associations between PAH456 exposure and PVD or SAD incidence, with adjustments for sex, weight, smoking parents (yes/no), pet in the home (yes/no), and distance of outdoor vehicular traffic (>100 m, 20–100 m, and <20 m). Linear regression was used to determine the associations between the ambient of PAH456 exposure and urine CRP or MDA levels. For concentrations below the method detection limit (MDL), half of the MDL was considered as censored data so that the dataset could be used for statistical analysis. Statistical significance level of the tests was set at *P* < 0.05, and analyses were performed using SPSS v.19.0 software (SPSS Inc., Chicago, IL, USA).

## 3. Results

### 3.1. Ambient Concentrations of PM2.5-Bound 4–6-Ring PAHs in Schools A and B

The GM, median, P25, P50, and P75 of ambient PM2.5-bound PAH456 concentrations are shown in Table 1. The values in the heating and nonheating seasons were compared for the two schools. The GMs of PAH concentrations ranged from 0.82 ng/m^3^ for DahA to 8.51 ng/m^3^ for BaA in the heating season and from 0.10 ng/m^3^ for DahA to 1.00 ng/m^3^ for BbFA in the nonheating season in school A, and from 1.03 ng/m^3^ for DahA to 11.59 ng/m^3^ for FLA in the heating season and from 0.12 ng/m^3^ for DahA to 1.17 ng/m^3^ for BbFA in the nonheating season in school B.

Ambient PM2.5-bound PHA456 levels were higher during the heating season as compared to the nonheating season (*P* < 0.01). Additionally, in the heating season, the GMs of BbFA, BkFA, BaP, DahA, and Σ10-PAHs in school B were higher than those in school A by 38%, 47%, 26%, 26%, and 39%, respectively (*P* < 0.05), with a marginally significant difference between schools for FLA, CHR, BaA, DghiP, and IcdP (*P* < 0.10). However, there was no difference in 4–6-ring PAH levels between schools in the nonheating season (*P* > 0.10).

### 3.2. Characteristics of the Study Population

The map of districts where the schoolchildren in this study attend school is shown in Figure 1. We compared demographic and exposure characteristics in the different educational settings (Table 2). The study included 82 (53.6%) and 65 (43.0%) male participants in schools A and B, respectively, with a mean age of 10 years. The mean weight ranged from 42.61 ± 10.62 kg (school A) to 40.46 ± 9.53 kg (school B), and the mean height ranged from 142.05 ± 9.11 cm (school A) to 139.76 ± 8.40 cm (school B).

We assessed exposure to environmental pollution through a questionnaire pertaining to whether there were smokers or pets in the household, whether an air purifier was used in the home, and the distance of outdoor vehicular traffic from the home. These were included in the model as categorical variables (Table 2). We found that 27.8% of subjects from school B reported smokers in the family, while 20.9% of children in school A were exposed to secondhand tobacco smoke from adults who smoked at home. Less than 20% of subjects (13.7% in school A and 16.6% in school B) reported the use of an air purifier at home. School A had a higher proportion of households with a pet than school B (*P* < 0.05). Furthermore, of the 153 subjects from school A, 51 (33.3%) reported the distance of outdoor vehicular traffic as >100 m, 43.8% as 20–100 m, and 22.9% as <20 m. In school B, 42.4% of students reported the distance of outdoor vehicular traffic as >100 m, 28.5% as <20 m, and 29.1% as 20–100 m (*P* < 0.05).

Urinary CRP concentration did not differ between the two schools, but urinary MDA concentration was higher in school B than in school A (*P* < 0.05).

### 3.3. Pulmonary Function Parameters in the Study Population

**Figure 2:**
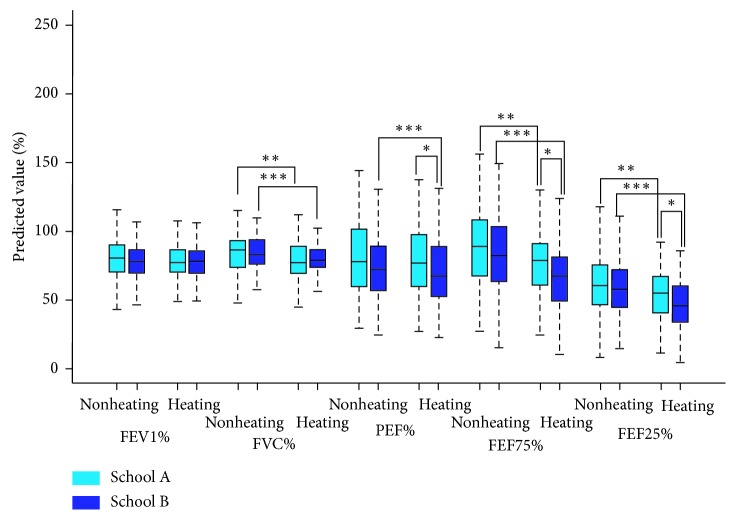
Descriptive statistics of lung function parameters among schoolchildren A and B. ^*∗*^*P* < 0.05 vs. schoolchildren A in the heating season; ^*∗∗*^*P* < 0.05 vs. schoolchildren A in the nonheating season; ^*∗∗∗*^*P* < 0.05 vs. schoolchildren B in the nonheating season.

The mean percentages of predicted FVC, FEV1, PEF, FEF25, and FEF75 values are shown in Figure 2. During the heating season, FVC%, FEF75%, and FEF25% were decreased in students of both schools, while PEF% was only lower in school B students during the nonheating season (*P* < 0.05). The parameters showing the greatest differences were FEF75% and FEF25% in the heating season, and the values of school B students (66.32% and 46.86%, respectively) were lower than those of school A students (78.83% and 54.60%, respectively) (*P* < 0.01).

### 3.4. Association between PM2.5-Bound 4–6-Ring PAHs Exposure and Rate of PVD and SAD

The PVD incidence in schools A and B was significantly higher during the heating season as compared to the nonheating season (school A: odds ratio [OR] = 2.696, 95% confidence interval [CI]: 1.672–4.348; school B: [OR] = 2.229, 95% [CI]: 1.384–3.590); however, for SAD, the occurrence rate was only higher in school B students ([OR] = 2.073, 95% [CI]:1.190–3.608; Table 3). During the heating season, the SAD incidence was approximately 11% higher in school B than in school A ([OR] = 1.919, 95% [CI]: 1.114–3.306); however, there was no difference in PVD incidence between the two schools.

We constructed a binary logistic regression model after adjusting for potential confounding factors, including sex, weight, smokers in family, pet in the home, and distance of outdoor vehicular traffic, to determine whether PAH456 exposure would increase the risk of PVD and SAD in schoolchildren from the heating to the nonheating season (Table 4). We found that 4–6-ring PAH levels were not correlated with the occurrence of PVD; however, the SAD occurrence was related to the exposure of BbFA, BkFA, BaP, and DahA, and the correlation coefficients for BbFA (OR = 1.452, 95% CI: 1.009–2.090) and DahA (OR = 2.705, 95% CI: 1.025–7.138) showed the strongest effect estimates in children from both schools.

### 3.5. Correlations between Urinary MDA or CRP Concentrations and Ambient PM2.5-Bound 4–6-Ring PAHs

As shown in Table 5, ambient PM2.5-bound BbF, BaP, and DahA were positively associated with MDA before or after adjusting for potential confounds such as sex, weight, smokers in the family, pet in the home, and distance of outdoor vehicular traffic. There was no correlation between ambient PM2.5-bound BbF, Bbk, BaP, and DahA and CRP level after adjusting for the confounding factors (model 3).

## 4. Discussion

The results of this pilot study reveal that airborne PM2.5-bound 4–6-ring PHA levels were significantly higher and the incidence of PVD and SAD in both schoolchildren was increased during the heating season. The concentration of airborne BbFA, BkFA, BaP, and DahA was associated with SAD incidence, and the correlation coefficients of BbFA and DahA were the highest effect estimates. We also found that ambient PM2.5-bound BbFA and DahA were associated with the level of the antioxidant stress factor MDA.

The concentration of PM2.5-bound Σ10-PAHs was about 10 times higher in the heating as compared to the nonheating season. This indicated that PAH emissions increase substantially during the winter. Moreover, exposure to ambient BaP (6.59 ng/m^3^ in school B), a representative atmospheric carcinogen, was approximately 10-fold higher than in London and New York during the months of December and January [23, 24]. The BaP level in the heating season reported in this study was six times higher than the national standard (1 ng/m^3^). This implied ambient PM2.5-bound PAH contamination is very serious in northeast China.

It is well known that exposure to polluted air can lead to airway inflammation and lung dysfunction, with children showing greater susceptibility to the detrimental effects of environmental PAHs. We found that lung function indexes, including FVC%, FEF75%, and FEF25%, were significantly lower in both schoolchildren during the heating season. We therefore evaluated the effect of exposure to PM2.5-bound PAH456 on the incidence of PVD and SAD in schoolchildren. The PVD and SAD incidence in schools A and B were higher during the heating as compared to the nonheating season. This indicates that coal consumption in the heating season is one of the risk factors for lung function parameters damage in schoolchildren. Additionally, SAD incidence was significantly higher in school B than in school A during the heating season. This is likely because school B is located in the Xiangfang district, which is a heavily industrialized area where ambient PAHs originate not only from coal consumption but also from local facility emissions. At present, epidemiological studies have shown that exposure to PAHs is associated with the occurrence of asthma, chronic obstructive pulmonary disease, and lung cancer [16, 25]. Meanwhile, a short-term increase in PAH456 level was associated with the risk of wheezing and a decrease in FEV1 among children in Fresno, California [15, 19]. Children living in a town where the air has high PAH levels had lower pulmonary function than those living in environments with less polluted air [26]. Additionally, higher urinary 1-hydroxypyrene level was associated with a decline in FEV1/FVC [27]. Our findings are consistent with the results of previous studies. Meanwhile, we found that PM2.5-bound PAH456 levels showed no correlation with the occurrence of PVD; however, the SAD occurrence was related to the exposure of BbFA, B(k)FA, B(a)P, and DahA. With increasing PAH456 exposure, BbFA and DahA have highest impact on SAD incidence. These results indicate that decreased lung function—which is mainly manifested as small airway dysfunction—is related to exposure to ambient PM2.5-bound BbFA and DahA. Since small airway dysfunction precedes the impairment of lung function, preventative intervention at an early stage can prevent respiratory diseases. At present, as a high concentration of BbFA in particle PAHs, DahA with high toxicity is less concerned. Most research studies have been conducted on reproductive toxicity of BbFA and the induction of skin tumors and lung carcinoma by DahA [28–30]. We provide here evidence of an association between ambient PM2.5-bound BbFA and DahA levels and SAD occurrence in schoolchildren.

PAHs are potent oxidants that contribute to inflammation and the pathogenesis of chronic diseases. In this study, we found that ambient PM2.5-bound BbFA, B(a)P, and DahA were positively associated with urinary MDA level before or after adjusting for potential confounds. Studies of adult participants of the National Health and Nutrition Examination Surveys found that urinary PAH levels were positively associated with serum markers of inflammation (CRP) and oxidative stress (gamma-glutamyl transferase) [31, 32], and both experimental and epidemiological evidence indicates that PAHs promote airway inflammation [33]. Exposure to PM2.5 and carcinogenic (c-) PAHs was linked to increased levels of fractional exhaled nitric oxide in healthy adults, and BbFA and BkFA were shown to have the highest effect estimates among c-PAHs [34]. According to the International Agency for Research on Cancer, high-molecular-weight PAHs are more likely to be carcinogenic to humans and cause greater oxidative stress damage than low-molecular-weight PAHs [35]. Our findings provide evidence that an increased oxidative stress burden may be one reason for the occurrence of SAD in schoolchildren exposed to PM2.5-bound PAH456 and possible subsequent development of pulmonary inflammation.

Our study had some limitations. Firstly, we did not obtain measurements of continuous levels of lung function parameters in schoolchildren (only two procedures were performed in 2017). Secondly, we measured only CRP to assess inflammation, which may not reflect the inflammatory state of the whole body. In future studies, we will increase lung function testing and examine the association between ambient PM2.5-bound PAH levels and changes in SAD incidence by panel study and evaluate additional parameters of systemic inflammation. Last but not least, the % predicted values of lung function parameters were lower than other studies. It may be the reason that Harbin is an industrial city in northern China with heavy air pollution and cold dry climate, leading to lower lung function parameters than other cities. We will further confirm these results in future experiments.

## 5. Conclusions

The results of this study indicate that exposure to PM2.5-bound PAH456 increases during the heating season in Harbin. Ambient PM2.5-bound BbFA and DahA have the highest impact on incidence of SAD. Oxidative stress may be one of the main reasons for the occurrence of SAD. This research showed that the identification of hazardous PM2.5-bound BbFA and DahA to health is crucial for preventing the respiratory-related diseases.

## Figures and Tables

**Table 1:** Comparison of ambient concentrations of PM2.5-bound PAH456 in schools A and B during heating and nonheating seasons (ng/m^3^).

	Heating season											Nonheating season										
	School A					School B					*P*	School A					School B					*P*
	GMs	M	P25	P50	P75	GMs	M	P25	P50	P75		GMs	M	P25	P50	P75	GMs	M	P25	P50	P75	
FLA	6.98	6.74	2.96	6.74	16.28	11.59	9.83	5.59	9.83	21.30	0.093	0.49	0.44	0.28	0.44	0.78	0.53	0.52	0.36	0.53	0.84	0.60
PY	7.30	7.41	3.29	7.41	18.48	9.84	9.11	4.56	9.11	18.50	0.154	0.30	0.32	0.19	0.32	0.70	0.40	0.55	0.27	0.55	0.99	0.31
CHR	8.34	8.23	4.36	8.23	15.95	10.74	10.36	5.79	10.36	17.75	0.067	0.65	0.74	0.39	0.74	1.65	0.61	0.74	0.46	0.74	1.22	0.32
BaA	8.51	8.69	4.01	8.69	17.70	11.20	11.30	5.18	11.30	21.95	0.060	0.47	0.39	0.20	0.39	1.78	0.44	0.39	0.24	0.39	0.88	0.48
BbFA	6.00	5.88	3.28	5.88	11.28	8.28	7.96	5.24	7.96	12.85	0.019^*∗*^	1.00	1.10	0.68	1.10	1.74	1.17	1.19	0.79	1.19	1.94	0.35
BkFA	3.79	4.65	1.65	4.65	10.30	5.57	6.80	2.74	6.80	10.59	0.042^*∗*^	0.93	1.01	0.59	1.02	1.61	1.00	1.06	0.75	1.06	1.76	0.24
BaP	5.23	5.31	2.82	5.31	9.84	6.59	6.46	3.50	6.46	11.65	0.034^*∗*^	0.53	0.47	0.29	0.47	1.02	0.54	0.47	0.28	0.47	1.14	0.21
DahA	0.82	0.89	0.38	0.89	1.60	1.03	0.99	0.52	0.99	2.15	0.037^*∗*^	0.10	0.11	0.05	0.11	0.17	0.12	0.11	0.06	0.12	0.27	0.45
BghiP	2.48	3.63	1.30	3.63	6.06	3.54	4.03	1.59	4.03	7.63	0.069	0.56	0.54	0.30	0.54	1.10	0.63	0.66	0.29	0.66	1.30	0.27
IcdP	2.66	3.50	1.12	3.50	7.19	3.40	4.47	1.35	4.47	8.90	0.075	0.49	0.44	0.26	0.44	0.96	0.57	0.53	0.27	0.53	1.22	0.13
Σ10-PAHs	54.72	52.25	26.89	52.25	115.24	76.32	75.71	39.49	75.71	125.81	0.046^*∗*^	5.96	6.44	3.79	6.44	10.22	6.69	6.56	4.42	6.56	9.87	0.37

GMs, geometric means; M, median; Σ10-PAHs, sum of 4–6-ring PAH concentrations; ^*∗*^vs. ambient PM2.5-bound 4–6-ring PAHs in school A during the heating season.

**Table 2:** Characteristics of the study population.

Variable	School A	School B	*P*
Number	153	151	
Sex (Male %)	82 (53.6%)	65 (43.0%)	0.066
Age (years (mean ± SD))	10.11 ± 0.94	10.37 ± 0.96	0.517
Weight (kg (mean ± SD))	42.61 ± 10.62	40.46 ± 9.53	0.087
Height (cm (mean ± SD))	142.05 ± 9.11	139.76 ± 8.40	0.283
Smoking in family (number (%))	32 (20.9%)	42 (27.8%)	0.085
Keeping pet at home (number (%))	35 (22.9%)	19 (12.6%)	0.019
Air purifier (number (%))	21 (13.7%)	25 (16.6%)	0.491
Distance of outdoor vehicular traffic			0.030
>100 m	51 (33.3%)	64 (42.4%)	
20∼100 m	67 (43.8%)	44 (29.1%)	
<20 m	35 (22.9%)	43 (28.5%)	
Urinary MDA (*μ*mol/L)	2.03 ± 1.05	2.57 ± 1.34	0.041
Urinary CRP (mg/L)	0.83 ± 0.31	1.01 ± 0.42	0.082

MDA, malondialdehyde; CRP, C-reactive protein.

**Table 3:** OR for the incidence of PVD and SAD in students of schools A and B in Harbin.

	Incidence of PVD (%)		Incidence of SAD (%)	
	School A	School B	School A	School B
Heating season	49.67^*∗*^	47.02^*∗*^	17.64	29.14^*∗*^^#^
Nonheating season	26.79	28.48	11.76	16.56
OR (95% CI)	2.696 (1.672–4.348)	2.229 (1.384–3.590)	1.607 (0.844–3.060)	2.073 (1.190–3.608)

OR, odds ratio; 95% CI, 95% confidence interval. ^*∗*^*P* < 0.05 vs. schoolchildren in the nonheating season; ^#^*P* < 0.05 vs. schoolchildren A in the heating season.

**Table 4:** Binary logistic regression between exposure to ambient PAH456 and the occurrence of PVD or SAD in schoolchildren^†^.

	Occurrence of PVD				Occurrence of SAD			
	School A		School B		School A		School B	
	Exp (OR)	95% CI	Exp (OR)	95% CI	Exp (OR)	95% CI	Exp (OR)	95% CI
FLA	0.988	0.967–1.010	0.947	0.856–1.047	0.937	0.903–1.044	1.026	0.992–1.134
PY	1.015	0.952–1.069	1.048	0.977–1.124	1.045	0.926–1.159	0.925	0.902–1.002
CHR	0.905	0.753–1.088	0.912	0.741–1.093	0.933	0.824–1.047	0.901	0.774–1.023
BaA	1.027	0.958–1.093	1.042	0.980–1.107	1.032	0.923–1.138	0.974	0.902–1.092
BbFA	1.094	0.853–1.305	1.187	0.920–1.532	1.324	1.088–1.612^*∗*^	1.452	1.009–2.090^*∗*^
BkFA	1.063	0.904–1.206	1.122	0.946–1.332	1.196	1.055–1.355^*∗*^	1.285	1.006–1.640^*∗*^
BaP	1.057	0.926–1.138	1.073	0.967–1.190	1.145	1.041–1.258^*∗*^	1.165	1.004–1.351^*∗*^
DahA	1.106	0.601–1.671	1.584	0.801–3.119	2.454	1.308–3.601^*∗*^	2.705	1.025–4.538^*∗*^
BghiP	0.868	0.668–1.127	0.83	0.689–1.070	1.072	0.935–1.216	1.016	0.904–1.127
IcdP	1.012	0.849–1.274	1.164	0.929–1.458	1.116	0.990–1.234	1.191	1.008–1.320^*∗*^
Σ10-PAHs	1.002	0.993–1.007	1.007	0.997–1.1017	0.959	0.904–1.020	1.015	1.000–1.029^*∗*^

^†^Adjusted for sex, weight, smokers in family, pet in the home, and distance of outdoor vehicular traffic. ^*∗*^*P* < 0.05.

**Table 5:** Correlations between urinary concentrations of MDA and CRP and ambient PM2.5-bound PAH456 in schoolchildren.

	BbFA	BkFA	BaP	DahA
*Model 1*				
MDA	0.226^*∗*^	0.215	0.231^*∗*^	0.244^*∗*^
CRP	0.214^*∗*^	0.191^*∗*^	0.202^*∗*^	0.210^*∗*^

*Model 2*				
MDA	0.212^*∗*^	0.194	0.217^*∗*^	0.223^*∗*^
CRP	0.186	0.176^*∗*^	0.194	0.195^*∗*^

*Model 3*				
MDA	0.173^*∗*^	0.161	0.177^*∗*^	0.194^*∗*^
CRP	0.169	0.164	0.163	0.185

Model 1, crude model; model 2, adjusted for pet in the home and distance of outdoor vehicular traffic; model 3, adjusted for sex, weight, smokers in family, pet in the home, and distance of outdoor vehicular traffic. ^*∗*^*P* < 0.05.

## Data Availability

The data used to support the findings of this study are available from the corresponding author upon request.
